# Infrared Laser-Assisted Extraction of Bioactive Compounds from *Rosa canina* L.

**DOI:** 10.3390/ijms26030992

**Published:** 2025-01-24

**Authors:** Andreia Alecu, Camelia Albu, Georgiana-Ileana Badea, Aurelia Alionte, Alin-Alexandru Enache, Gabriel-Lucian Radu, Simona-Carmen Litescu

**Affiliations:** 1 Centre of Bioanalysis, National Institute of Research and Development for Biological Sciences, 060031 Bucharest, Romania; andreia.alecu@incdsb.ro (A.A.); camelia.albu@incdsb.ro (C.A.); georgiana.badea@incdsb.ro (G.-I.B.); aurelia.alionte@incdsb.ro (A.A.); lucian.radu@incdsb.ro (G.-L.R.); 2 S.C. Apel Laser S.R.L., 077135 Mogosoaia, Romania; alin.enache@apellaser.ro

**Keywords:** extraction technologies, infrared laser radiation extraction, accelerated solvent extraction, *Rosa canina* L., polyphenolic compounds, lipophilic compounds

## Abstract

The extraction of bio-compounds from medicinal plants provides opportunities for using the plant extract for health benefits. *Rosa canina* L. is considered a “natural superfood”, and the valorization of its active compounds requires an extraction technique that ensures a suitable extraction yield while preserving the compounds’ activity. In our study, infrared laser irradiation (IRLIR) technology was used for the first time in the bioactive compound’s extraction from *Rosa canina* L. Different solvents (water–ethanol, hexane–ethanol) and different extraction times were tested to obtain a high extraction yield. Chromatographic and spectrophotometry methods were used to monitor the profile of bioactive compounds and the antioxidant activity of the extracts. The results obtained for IRLIR were compared with those obtained by accelerated solvent extraction (ASE), an advanced extraction method. The IRLIR technology proved to be a more reliable analytical tool for the extraction of (+)-catechin, gallic acid, and lutein. In addition, a richer extract formula was obtained by IRLIR extraction with respect to ASE, with the IRLIR process ensuring a short extraction time, low volume of the extraction solvent, low energy consumption, and a less expensive device.

## 1. Introduction

Suitable extraction of bioactive compounds from plants and fruits is the first important step and possibly the most important step for their subsequent use and, of course, for their assessment. It is a continuous development of the extraction field [1,2] to develop innovative strategies for extraction techniques that provide a cost-effective production of high-quality extracts being developed. The main purpose is to achieve a high extraction yield of the natural compounds, maintaining their bioactivity to ensure minimal energy consumption and, last but not least, minimal process stages are desired. Additionally, according to environmental sustainability, there is a need for strategies that consist in reducing the amount of solvent/raw materials used and generate of a minimal amount of waste, which must be safe for environment and living beings, avoiding soil/water degradation and/or contamination [3,4].

There is no standard method for bioactive compounds’ extraction from vegetal materials, so any new or modified technique that can contribute to improving the extraction yield of one or more active principles is of interest. The new trend in the extraction of bioactive compounds from plants is to use advanced extraction techniques to enhance the process efficacy, like cavitation-based extraction techniques (e.g., ultrasound-assisted extraction (UAE), microwave assisted extraction (MAE)) or accelerated solvent extraction (ASE). When bioactive compounds have to be recovered in a higher yield and with a high degree of specificity from vegetal material, supercritical fluid extraction (SFE) and even enzyme-assisted extraction (EAE) are employed. All these techniques overcome the limitations of traditional procedures (e.g., Soxhlet extraction, maceration, percolation, infusion, decoction), such as laborious protocols, large volumes of solvents, significant amount of waste, excessive time consumption, and lack of extraction selectivity [1,2,3,4]. In the last few years, the laser irradiation (LIR) technique has caught the attention of researchers in the extraction of bioactive compounds from medicinal plants [5,6]. In the beginning, LIR was mainly used to increase the growth rate and the production yield for secondary metabolites in plants, either as a result of associated stress or as a result of increasing the amount of plant biomass. Different types of lasers were used, namely, 630–660 nm (He–Ne, GaAlAs, diode), 515–532 nm (Nd:YAG; Ar), and 410 nm (diode), for the generation of red, green, and blue light that were applied to seeds and/or plants for bio-stimulation [7,8]. The type and applied power of laser affect the composition profile of occurring active compounds, like carotenoid, chlorophyll [8], free fatty acids, and free amino acids [9]. LIR was also applied at 343 nm, 750 nm, 800 nm, 1064 nm, and 1550 nm for cell permeabilization and molecular delivery [10,11]. Other researchers used LIR at 488 nm, 514 nm, 532 nm, 552 nm, 660 nm, 785 nm, 1270 nm, and 1550 nm for minerals, trace elements, and bioactive compound extraction from medicinal plants [5,6].

LIR of a solid sample in a liquid reaction environment causes various effects. One of the effects induced by LIR is the thermal effect resulting from the release of the laser energy, which leads to heating of the exposed area. The increase in temperature causes membrane permeability, which improves the compound exchange. The thermal effects are small when the irradiation time is short or a pulsed laser is used [11] but they intensify with laser energy input [12] and wavelength [13]. The infrared (IR) LIR conducts a higher thermal effect, particularly when the extraction medium contains water. Water molecules better absorb the energy emitted by the IR laser [12,13] and convert it into heat. Following the collision between the laser radiation and the extraction medium, there is also an increase in pressure, forming bubbles [14]. The bubbles’ cavitation and lifetime depend on the spot size of the laser beam [15] and the laser energy [14]. Other LIR effects are the disruption of the cell wall [12]. Cell disruption is enhanced by using a continuous laser system [12]. The wavelength of the laser directly affects the penetration depth of the radiation. Therefore, a higher wavelength, especially IR wavelength, results in greater laser penetration [13,16]. LIR was carried out to accelerate the reaction rate of the extraction process, facilitating the mass transfer of the target compounds into the extraction environment. Previous studies have shown that the phenolic content increased up to 15.5% in aqueous extracts of *Plantago lanceolata* L. *folium* [5], and the (iso)flavonoid compounds increased by 2–3 times in the hydroalcoholic extracts of *Medicago sativa* [6].

Another promising alternative and likely frequently used method for bioactive compound extraction from vegetal materials is accelerated solvent extraction (ASE) [17,18]. ASE is an automated extraction technique that uses organic solvents at high temperatures (up to 200 °C) and pressures (1500 PSI). The elevated temperature and the high pressure increase the efficiency of extracting analytes from the sample and reduce extraction times and solvent consumption. The extraction is more effective compared to conventional extraction methods due to increasing the solubility of bioactive compounds in the extraction solvent due to a greater speed of diffusion rates, decreasing the solvent viscosity and weakening solute–matrix interactions, allowing the analytes to be easily extracted from the sample matrix [6,19,20]. However, the choice of the extraction conditions (temperature, time) must be chosen carefully to not affect the stability of the target compounds. Higher temperatures and long extraction times can produce thermal degradation of bioactive compounds and evaporate the extraction solvent. The use of diatomaceous earth that fills the sample cell offers the advantage of a homogeneous distribution of the sample, prevents the aggregation phenomenon, and increases the contact surface between the extraction solvent and the sample. In addition, sorbent materials can be used to eliminate undesirable compounds [20]. Another advantage of the technique consists in using the same sample for the removal or extraction of certain bioactive compounds using different extraction methods without introducing additional steps in the extraction process.

In the last decade, the growing importance of natural compounds was noticed due to greater availability and less adverse side-effects compared to synthetic compounds. Plant extracts are a rich source of bioactive compounds and have found applications in pharmaceutical, food, and cosmetic industries [21]. Plant extracts also have the advantage that they can be used in models for innovative drugs or alternative products [22].

The *Rosa* spp. family is one of the most diversified types of plants, containing up to 200 varieties [23]. The *Rosa canina* L. variety is one of the most important species in the economical, ecological, and therapeutic point of view [24,25]. The *Rosa canina* L. contains a vast number of bioactive compounds, like flavanols (catechin and epicatechin and their derivates), flavonols (quercetin with its derivates, kaempferol and its derivates), flavonones (luteolin derivates, apigenin derivates), phenolic acids (gallic acid, ellagic acid, chlorogenic acid, ferulic acid, p-coumaric acid), anthocyanins, vitamins (C, B, K, P, A, E, D), carotenoids (β-carotene, lycopene, rubixanthin, lutein, zeaxanthin), fatty acids (linoleic, oleic, palmitic, stearic), and minerals (Na, K, Ca, Mg, Mn, Fe, Cu, Zn, P, S, Se) [22,23,26,27,28,29,30]. The wide composition profile of the *Rosa canina* L. confers properties such as anti-oxidative, anti-inflammatory, anti-cancerogenic, anti-microbial, and diuretic [23,31,32], ensuring beneficial effects that maintain human health and prevent or treat illness. The composition profile of the bioactive compounds in a specific plant is highly dependent on growing conditions, soil, the amount of precipitation per season, and the harvesting period, and, accordingly, even the efficacy and the recovered quantity of specific active compounds is influenced by these factors [5,28,32]. The *Rosa canina* L. can be used at any stage of its maturity and can be consumed as beverages (tea, wine, juice, syrup) or food (jam, jelly, soup, cake, snacks). Additionally, it can be used as an ingredient in supplements or cosmetics [29,32,33,34,35]. *Rosa canina* L. extracts are obtained through classic (maceration, infusion) and advanced (ultrasound-assisted extraction (UAE)) extraction technologies. The choice of extraction solvent plays a significant role in the yield and quality of plant extracts, as well as in the environmental and economic aspects of the extraction process. As extraction solvents, the most commonly used for polyphenolic compound extraction are ethanol and water due to their polar nature and non-toxicity. Variate mixtures of ethanol–water (volume: volume, v:v, 100:0; 80:20; 70:30; 50:50; 0:100) were tested [24,30,31,35,36]. When only water was used as a solvent extract, the content of impurities increased [24], and when only ethanol was used as a solvent extract, the content of polyphenols decreased significantly [36]. While non-polar organic solvents (hexane, chloroform, dichloromethane) are commonly used for carotenoid extraction, the increasing focus on green chemistry has led to the exploration of safer (ethanol, oils) or more sustainable alternatives (various mixtures between polar and non-polar) [37,38,39]. There is a continuous process to optimize extraction methods balancing efficiency, safety, and environmental impact.

The aims of our study were to investigate the potential of a new extraction technique, IRLIR, to recover bioactive compounds from *Rosa canina* L. and to assess IRLIR capability to preserve the antioxidant activity of the extracts when compared to a highly efficient extraction technique, ASE. Therefore, the performance of the IRLIR using near-infrared (NIR) laser radiation was assessed at three wavelengths, 1064 nm, 1270 nm, and 1550 nm, and two energy powers, 150 mW and 300 mW. The IRLIR technique was used for the first time on *Rosa canina* L. and, most importantly, it is a proactive approach to develop a technology supporting environmental sustainability. The idea of using IRLIR-assisted extraction was born from the supposition that by weakening the H bond between the active compounds from *Rosa canina* L. and phospholipid from the vegetal cell wall, the release of bioactive compounds is facilitated. The H bond with phospholipids from cell membranes is due to phenolic -OH groups, which are mainly H donors. The experiments were carried out on *Rosa canina* L. due to its usefulness in the food and cosmetic industry.

## 2. Results and Discussion

The IRLIR extraction was performed in a flexible way, using appropriate mixtures of solvents and different source frequencies in order to enhance extraction specificity.

For polyphenolic compounds, ethanol and water were used. The choice of optimal solvent ratios as the best extraction solvent was made considering the different solubilities of the polyphenol compounds, in order to achieve a high extraction yield of these compounds. Based on the targeted compounds’ solubility and considering the preliminary results obtained while building the device and developing the extraction process, two mixtures of water–ethanol, 1:4 (*v*/*v*) and 4:1 (*v*/*v*), were finally used in our tests. Since IRLIR technology is new in this domain, to achieve a high extraction yield of the bioactive compounds, different operating parameters, including the laser frequency and power, needed to be optimized. Considering the preliminary results attained when all five available laser sources (532 nm, 655 nm, 1064 nm, 1270 nm, and 1550 nm) were tested on vegetal materials with high contents of polyphenols and carotenoids, three laser sources (1064 nm, 1270 nm, and 1550 nm), two laser powers (150 mW and 300 mW), and different extraction times (5 and 15 min) were chosen to determine the optimal parameters to establish the best conditions for bioactive compound extract formulation.

For the lipophilic compounds, extraction was performed using a laser with a wavelength of 1064 nm, which proved to be more efficient for non-polar active compounds [40], using laser powers of 150 mW and 300 mW and an extraction time of 5 and 15 min, respectively. An ethanol–hexane mixture was used as the extraction solvent.

### 2.1. Composition Profile of Formulated Extracts

#### 2.1.1. Polyphenol Compound Analysis of *Rosa canina* L. Extracts

The efficiency of different IRLIR parameters in enhancing the extraction yield was assessed based on the results of the HPLC-MS analyses obtained when monitoring the polyphenol compounds profile. Some of the performance characteristics of the HPLC-MS method were determined. The calibration curves were attained for six different concentrations in the domain of 1–100 µg mL^−1^. The limit of quantification (LoQ) and the limit of detection (LoD) were calculated as 10 and 3 times, respectively, the signal-to-noise ratio. The values obtained for the range of response, correlation coefficients (R), LoQ, and LoD confirm that our method is suitable for the analysis of polyphenolic compounds in *Rosa canina* L. extracts samples (Table 1).

**Figure 1:**
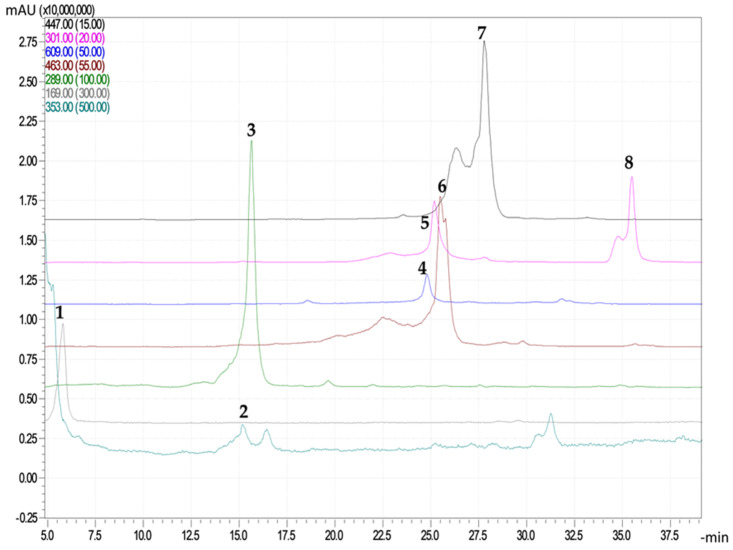
Chromatograms obtained for *Rosa canina* L. extract after IRLIRE in 80% ethanol (1—gallic acid, [M − H]^−^ 169; 2—chlorogenic acid, [M − H]- 353; 3—(+)-catechin, [M − H]- 289; 4—rutin, [M − H]- 609; 5—ellagic acid, [M-H]- 301; 6—quercetin 3-β-D-glucoside, [M − H]- 463; 7—quercitrin, [M − H]- 447; 8—quercetin, [M − H]- 301) by HPLC-MS.

The major polyphenolic compounds (Table 2 and Table 3) identified by HLPC-MS from *Rosa canina* L. extracts obtained under different conditions through the IRLRI technique were as follows: (+)-catechin (flavanols), rutin, quercetin, quercetin 3-β-D-glucoside and quercitrin (flavonols), and gallic acid, ellagic acid, chlorogenic acid (polyphenolic acids) (Figure 1).

The composition profiles and concentrations of the polyphenolic compounds from hydro-alcoholic extracts of *Rosa canina* L. analysed through the HPLC-MS method proved to be greatly affected by the extraction solvent. It was noticed that the use of the IRLIR technique along with 20% ethanol resulted in a higher amount of polyphenolic compounds compared to 80% ethanol. The achieved profile was catechin > gallic acid > quercetin 3-β-D-glucoside > ellagic acid > quercitrin > chlorogenic acid > quercetin > rutin. The content of gallic acid and catechin found in our study through the IRLIR technique was higher (132 µg g^−1^ and 173 µg g^−1^) than that found by other methods. In the *Rosa canina* L. extracts obtained by maceration with or without stirring, a gallic acid content of 2.21 µg g^−1^ of dried weight of extract from 0.3 µg g^−1^ dry fruit was found [41,42]. As is easily noticeable, our method led to an increase of around 2 magnitude order of the amount of gallic acid in the formulated extract. If we consider catechin, reported methods indicate a content of 134.75 µg g^−1^ with stirring and of 11.9 µg g^−1^ without stirring during maceration [41,42], while in our methods yield to an amount of the same magnitude order as that obtained from dry fruit. When compared to other published data about extraction based on cavitation phenomena, such as UAE [35], our IRLIR results led to less effective recovery of the gallic acid and catechin amount but to a higher abundance of other polyphenols, thus allowing better efficiency of the obtained extracts. When the extraction solvent was 80% ethanol, the polyphenolic profile was different: ellagic acid > quercetin 3-β-D-glucoside > quercitrin > chlorogenic acid > quercetin > catechin > gallic acid > rutin. As can be seen from Table 2 and Table 3, the IRLIR technique might be used to perform a sort of selective extraction directly in the extraction reactor, thus reducing the efforts for specific separation. A high extraction yield for gallic acid was achieved when using 1064 nm and 150 mW laser characteristics and an extraction time of 15 min and 20% ethanol, while the ellagic acid was best extracted at 1064 nm, laser power 300 mW, and extraction time of 5 min in 80% ethanol. The achieved higher yield of extraction of gallic acid, (+)-catechin, and chlorogenic acid in 20% ethanol is based on the specific solubility of the compounds and their polarity, while better extraction of quercetin, its derivatives, and ellagic acid in 80% ethanol is due to the solubility characteristics and lactone ring presence. A higher laser power increases both the energy focalized on the cell wall and the water decomposition; therefore, a certain degree of an undesirable degradation process due compound degradation as result of oxidation or other chemical reactions (20% ethanol involves a higher amount of water in the extraction solvent) could occur, thus a certain decrease in bioactive compound concentration is registered.

To verify that IRLIR is a reliable, robust, and efficient tool for the extraction of polyphenol compounds from *Rosa canina* L., we compared the results with those acquired by an advanced extraction technique, namely, ASE, a technique more frequently used when dealing with the extraction of polyphenolic compounds from plants and fruits [6,19]. The experiments were performed using the same extraction solvent applied in IRLIR. The results of the HPLC-MS analyses gained for *Rosa canina* L. extracts by ASE are shown in Table 4.

In the case of the ASE technique, the polyphenolic profile was similar, regardless of the used extraction solvent, and varies in order: ellagic acid > quercetin 3-β-D-glucoside > quercitrin > catechin > quercetin > chlorogenic acid > rutin > gallic acid. A higher percentage of water and a low percentage of ethanol led to an increase in polyphenolic compounds extracted from *Rosa canina* L. regardless of the extraction technology used (IRLIR or ASE). However, there are differences between the compositions of the polyphenolic compounds resulting from the two extraction techniques used. The gallic acid was extracted in very low concentrations (only 9.52 mg g^−1^) through ASE technology, but ellagic acid and quercetin derivates were in higher concentrations.

#### 2.1.2. Lipophilic Compound Analysis of *Rosa canina* L. Extracts

**Figure 2:**
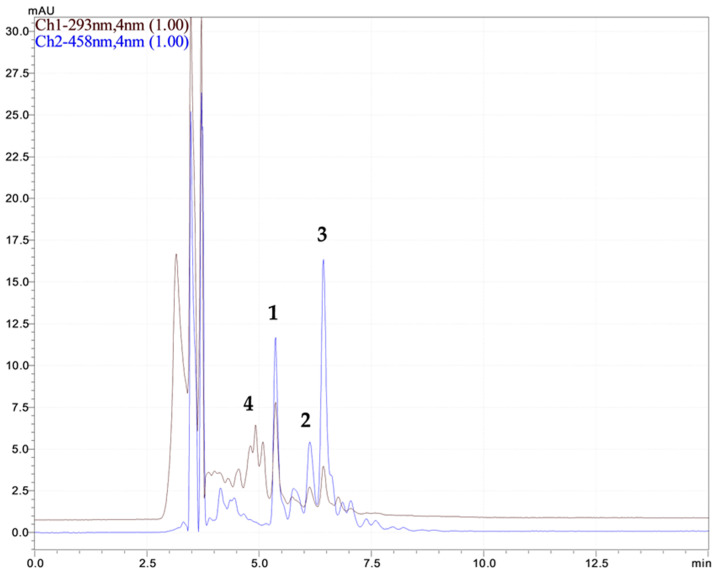
Chromatograms obtained for *Rosa canina* L. lipophilic extract after IRLIRE in ethanol–hexane mixture (1—lycopene; 2—lutein; 3—β-carotene; 4—α tocopherol) by HPLC-DAD.

The identification of the compounds was performed by comparing retention times and UV–Vis spectral data with those of the corresponding standards (Figure 2). Furthermore, the cis-isomers of the carotenoids were recognized based on their distinctive spectral patterns. [43,44]. All the cis-isomers of the carotenoids were quantified based on the calibration curve of the all-trans-β-carotene standard due to the similarity of their extinction coefficients. Several performance characteristics of the used analytical method are given in Table 5 below.

In assessing the composition profile of the lipophilic extracts by HPLC-DAD, it was proven that they are rich in carotenoids and tocopherol. The obtained concentrations of tocopherol, beta-carotenes, lutein, and lycopene are given in Table 6.

It was noticed that higher amounts of the lipophilic compounds extracted were found when 150 mW laser power was used for 15 min. The results were compared with those obtained using ASE extraction in the same solvent mixture, wherein the formulated extracts were quantified as 376.29 µg g^−1^ α-tocopherol, 1791.38 µg g^−1^ β-carotene, and 1413.42 µg g^−1^ lycopene; no lutein was extracted. It could be concluded that a richer extract formula was obtained by IRLIR extraction with respect to ASE. If we compare our results with those reported in the literature [45,46,47], we may conclude that our IRLIR method proved to lead to a higher extraction yield for β-carotene and lycopene (two magnitude fold order higher) and better recovery of α-tocopherol.

### 2.2. Assessment of Formulated Extract Efficacy as Antioxidants

#### 2.2.1. The Antioxidant Capacity of the *Rosa canina* L. Polyphenolic Extracts

The antioxidant capacity was assessed through reduction in the cation radical of 2,2-azinobis(3-ethylbenzothiaziline-6-sulfonate) (ABTS) in the presence of bioactive compounds with antioxidant properties. The decrease in the recorded absorbance for the blue-green complex formed between ABTS+∙ and bioactive compounds is proportional to the antioxidant capacity of the sample. An effective bioactive compound or a high content of this conduct also led to a greater decrease in intensity.

The TEAC values of the *Rosa canina* L. extracts obtained by IRLIR are presented in Table 7. When 20% ethanol was used, the results ranged in the 17,492–23,619 µmol g^−1^ domain. The highest TEAC value was attained when 1550 nm laser wavenumber, 150 mW laser power, and 15 min extraction time was tested. An increase in the laser power to 300 mW led to a decrease of up to 15% of the antioxidant capacity that is affected by the laser wavenumber and extraction time. At a low laser power, the TEAC values varied by 5% regarding different extraction times. At a high laser power, the TEAC values increased up to 20% depending on laser wavenumber. When 80% ethanol was used, the obtained results ranged from 3330 to 5238 µmol g^−1^. The highest value was achieved when 1064 nm laser wavenumber, 150 mW laser power, and 5 min extraction time was tested. An increase in the laser power showed a discrepancy in the antioxidant capacity up to 30%. These differences can be ascribed to re-structuration of the bioactive compounds due to the laser characteristics [48].

From the results obtained for the antioxidant activity of the *Rosa canina* L. extracts (Table 7), it was noted that the use of 20% ethanol as an extraction solvent leads to samples with an antioxidant capacity about 5 times higher than the samples extracted with 80% ethanol due to the specificity of the active compounds’ solubility. The antioxidant capacity depends not only on the concentration of the antioxidant compound but also on its structure, especially by number of hydroxyl groups. Antioxidant activity is also influenced by synergistic or antagonistic interactions between different polyphenol compounds, as well as their interactions with other components of the *Rosa canina* L. extracts matrix. It is known that gallic acid and catechins, which were extracted in high amounts, are powerful antioxidants providing protection against hydroxyl (HO∙), superoxide (O_2_∙^−^), peroxyl (ROO∙), and hydrogen peroxide (H_2_O_2_) [49,50].

**Figure 3:**
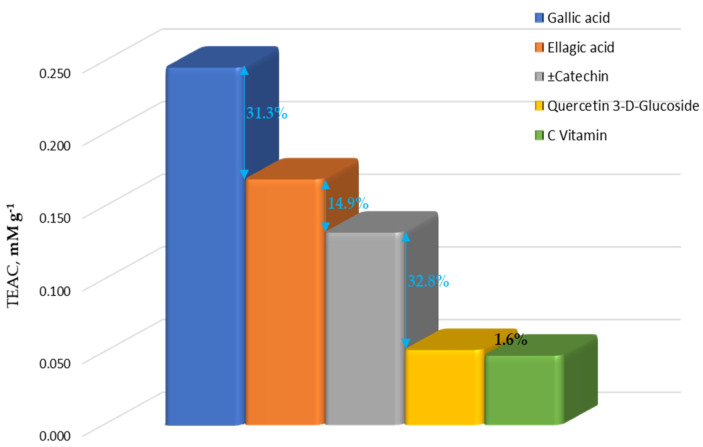
The antioxidant capacity of standard polyphenolic compounds in 20% ethanol.

In the situation when we have no influences of the sample matrix, gallic acid shows the highest antioxidant activity in 20% ethanol, the optimal extraction solvent. The order of antioxidant capacity of standard polyphenolic compounds, as main compounds found in *Rosa canina* L. extracts, is gallic acid > ellagic acid > catechin > quercetin 3-D-glucoside > C vitamin (Figure 3). This confirms the fact that the samples with a high content of gallic acid and catechin show higher antioxidant activity.

It should be mentioned that since one of the main constituents of hydroalcoholic extracts from *Rosa canina* L. is vitamin C [30], to avoid any artifacts related to antioxidant efficiency results, both quantification of the amount of vitamin C extracted from the vegetal material and evaluation of TEAC value that is ascribable to vitamin C were performed. For the 3.13 mg g^−^^1^ of vitamin C found in the *Rosa canina* L. extract, the TEAC value was 642 µmol g^−^^1^ for the optimal IRLIR parameters, namely, the 1064 nm source, 150 mW laser power, and 20% ethanol. Therefore, if the synergistic effect is not considered, it might be underlined that the contribution of vitamin C is around 3%; thus, the potential occurrence of vitamin C in a formulated extract does not significantly affect the TEAC value.

Due to the fact that when 20% ethanol is used, the content of bioactive compounds with antioxidant activity is higher, the oxygen radical absorbance capacity (ORAC) assay was applied only for this extraction solvent. The results are presented in Table 8.

In the context of the ORAC assay, it can be stated that a low laser power (150 mW) leads to obtaining higher antioxidant capacity values for the *Rosa canina* L. polyphenolic extracts. This may be attributed to the preservation of the antioxidant properties of the bioactive compounds found in the extracts.

The TEAC values obtained through ASE are presented in Table 9. The results confirm the HPLC-MS data, namely, that the mixture of ethanol–water = 1:4 (*v*:*v*) leads to better extraction yield.

Compared with the best results obtained by the IRLIR technique, the antioxidant capacity of these extracts is 4 times lower when 20% ethanol was used and by 37% when 80% ethanol was used, thus making possible the assertion that IRLIR extraction ensures better preservation of structural integrity of the hydrophilic bioactive compounds.

#### 2.2.2. The Antioxidant Capacity of the *Rosa canina* L. Lipophilic Extracts

The antioxidant capacity of lipophilic extracts from *Rosa canina* L. was studied using the ORAC assay, a reliable method to evaluate the antioxidant potential of these compounds, and using vitamin E as a reference compound instead of Trolox. The antioxidant capacity (AC) values are given in Table 10 for IRLIR extraction, while the value obtained when the extraction was carried out by ASE was 8288 ± 35 µmol g^−^^1^.

## 3. Materials and Methods

### 3.1. Reagents

All the reagents used in this study were of analytical grade. Ethanol, 2,2′Azobis(2-methylpropionamidine) dihydrochloride (AAPH), 2,2-azinobis(3-ethylbenzothiaziline-6-sulfonate) (ABTS), ascorbic acid (vitamin C), and polyphenol compounds ((+)-catechin, quercetin, quercetin 3-D-glucoside, quercitrin, rutin, chlorogenic acid, acid 6-hidroxi-2,5,7,8-tetrametillchroman-2-carboxilic (Trolox), monopotassium phosphate, dipotassium phosphate, sodium borate, ethanol, and hexane) were purchased from Sigma-Aldrich GmbH (Steinheim, Germany). Gallic acid, ellagic acid, sodium dodecyl sulfate (SDS), and fluorescein were purchased from Fluka (Sigma-Aldrich GmbH, Steinheim, Germany). Acetonitrile and formic acid were purchased from Riedel-de Haen (Seelze, Germany) and Merck (Darmstadt, Germany), respectively. All the solvents were of chromatographic purity. The Elix 3 (Millipore, Darmstadt, Germany) system was used to obtain ultra-pure water.

### 3.2. Preparation of Rosa canina L. Extracts

The plant material, *Rosa canina* L., presented as dry fruit without seeds, was a marketable product from Larix (Sovata, Romania). The vegetal samples were ground and homogenized to powder using a Retsch GmbH knife mill, GRINDOMIX GM200 (Haan, Germany) for 1 min at 5000 rpm. All the plant extracts were prepared in a similar manner, namely, 10 g of the *Rosa canina* L. sample were mixed with extraction solvent, and for the polyphenols, a mixture of ethanol–water, 1:4 *v/v* and 4:1 *v*/*v*, respectively, were used. For the lipophilic extract, a mixture of ethanol–hexane, 4:3 *v*/*v* was used. Subsequently, there were different extraction procedures at different temperatures and pressures depending on the technique used. All the obtained *Rosa canina* L. extracts were stored in the dark at 4–6 °C until HPLC and antioxidant activity analyses. Prior to HPLC analysis, the samples were filtered into HPLC vials with a 25 mm, 0.2 µm syringe-driven filter unit PTFE (Agilent, Beijing, China) and injected into the HPLC system.

#### 3.2.1. *Rosa canina* L. Extracts Obtained by IRLIR

The solid–liquid mixture was subjected to radiation using an APE WaveScan Laser with a diode DPSSL spectrometer (S.C. Apel Laser S.R.L., Mogosoaia, Romania) with IR laser emitting at different wavelengths (1064 nm, 1270 nm, and 1550 nm). The characteristics of the used sources are given in Table 11.

The reaction chamber was developed and realized by S.C. ApelLaser S.R.L. The volume of the solvent used for the IRLIR method was 50 mL. The operating parameters are presented in Table 12. The laser light passed through a beam expander that covered the sample recipient to increase the contact surface. During the extraction procedure, the sample was continuously stirred using a magnetic stirrer, model LLG-uniSTIRRER 2, from LLG Labware—RoHS compliant. After the extraction time, 5 and 15 min, the samples were filtrated using filter paper.

#### 3.2.2. *Rosa canina* L. Extracts Obtained by ASE

ASE was performed using optimized in-house-developed methods [6] to extract polyphenols and lipophilic bioactive compounds, respectively (carotenoids and tocopherols). The vegetal powder was mixed with diatomaceous earth until good homogenization was achieved. The obtained mixtures were used to fill the extraction stainless-steel cells, which have a capacity of 100 mL. Thermo Scientific filters (glass fiber for organic solvent, and cellulose for aqueous solvent) were placed at the bottom of the cells. For the polyphenolic extracts, the extraction was performed using an automated Thermo Scientific™ Dionex™ ASE™ 350 Accelerated Solvent Extractor (Waltham, MA, USA) at 50 °C, at approximately 1600 PSI, 3 cycles for 5 min. The system was purged with nitrogen, and fresh extraction solvent was pumped through the sample and the entire system. The energy consumption was 500 VA, maximum. The extracts were collected in glass vials with Teflon septa and kept at 4–6 °C until analyses. The extraction solvent was added automatically by an ASE extractor. For the polyphenolic extracts, the solvents used were 20% ethanol and 80% ethanol, respectively, while for the lipophilic extracts, a mixture of ethanol–hexane 4:3 (vol/vol) was used.

### 3.3. Methods

#### 3.3.1. HPLC-MS Analysis of *Rosa canina* L. Polyphenolic Extracts

HPLC analysis was performed using a Shimadzu system (Shimadzu GmbH, Kyoto, Japan) consisting of a DGU-20A degasser, a SIL-20AC autosampler, a CTO-20A column oven, two LC-20AD pumps, a mass spectrometer detector, LCMS-2010 with an ESI interface, negative ionization mode, and the following parameters: CDL temperature, 200 °C; interface temperature, 250 °C; heat block temperature, 200 °C; nebulization gas (N2) flow rate, 1.5 L min^−1^; detector voltage, 1.8 kV; and interface voltage 4 kV.

The identification and quantification of the polyphenol compounds were performed with a method previously developed by our team [51]. A Kromasil column 100-3.5-C18 (Nouryon Kromasil, Sweden) was used, and the mobile phase, pH = 3.00, consisted of formic acid in water (solvent A) or in acetonitrile (solvent B). The elution was performed using a mobile phase gradient and a flow rate gradient to achieve the efficient separation of the active principles, with the gradient sequences being as follows: 0.1 mL min^−1^ from 0 to 5 and 0 to 20 min for 5–30% solvent B; 0.2 mL min^−1^ between 5.01 and 15 min, 0.1 mL min^−1^ from 15.01 to 35 min; 20.01–40 min for 30% solvent B; 0.2 mL min^−1^ between 35.01 and 60 min; 40.01–50 min for 50% solvent B; 50.01–52 min for 50–5% solvent B; 0.1 mL min^−1^ from 60.01 to 62 min and 52.01 to 62 min for 5% solvent B.

For quantitative analysis, the selected ion monitoring (SIM) mode was utilized, and the next [M − H]- was 169, 289, 301, 353, 447, 463, and 609, corresponding peaks of the polyphenols compound fragment ions.

#### 3.3.2. HPLC-DAD Analysis of *Rosa canina* L. Lipophilic Extracts

The HPLC analysis of lipophilic compounds was performed using a SHIMADZU 20AD HPLC system (Shimadzu GmbH, Kyoto, Japan) with a C18 Kromasil (Nouryon Kromasil, Sweden) chromatographic column, 250 × 4.6 mm, 5 μm. The optimal conditions for the mobile phase and elution are as follows: chromatographic column temperature of 35 °C; mobile phase flow rate of 0.7 mL min^−1^; mobile phase component ratio A:B = 20:80; mobile phase composed of component A (acetonitrile) and component B (dichloromethane–methanol (60:40)); injection volume of samples was 20 μL; monitored wavelengths: 293 nm for α-tocopherol and 458 nm for β-carotene.

#### 3.3.3. Antioxidant Capacity

The antioxidant capacities of the *Rosa canina* L. extracts were assessed by the Trolox equivalent antioxidant capacity (TEAC) protocol [51] using a Thermo Evolution 260 Bio Spectrophotometer (Thermo Fisher Scientific, Shanghai, China). The method is based on reduction in cationic radical, ABTS+∙, which is soluble in extraction solvent (water–ethanol mixture) and permits assessment of the antioxidant capacity for hydrophilic compounds. Additionally, ABTS+∙ is a stable radical and leads to reproductible results that are expressed in Trolox equivalents per amount of extract taken into the work [52]. The samples were diluted appropriately with solvent. The decrease in the ABTS+∙ absorbance was monitored at 735 nm after 3 min due to the fact that the reaction between antioxidants and free radicals takes place quickly.

An ORAC assay was performed to assess the antioxidant capacity of the hydrophilic and lipophilic extracts using an FP-8500 Spectrofluorometer (JASCO Corporation, Tokyo, Japan). The protocol consists of measurements performed at an excitation wavelength of λex = 490 nm and an emission wavelength of λem = 514 nm. First, measurements of the blank were taken (phosphate buffer, fluorescein, and peroxyl radical production (obtained from AAPH)), with the fluorescence emission being recorded over a reaction time of 30 min. After that, the measurements were taken for the reference compound (standard), Trolox, and, finally, the samples. The antioxidant capacity was calculated according to a previously reported method [52]. When lipophilic extracts are assessed for antioxidant efficacy, the ORAC test is applied using micelles with SDS–boric acid–buffer (1.5:1.5:7 = vol:vol:vol), and the antioxidant capacity is calculated using vitamin E as a reference compound instead of Trolox.

#### 3.3.4. Statistical Analysis

Statistical analysis was carried out using Microsoft Office Excel (Office 365, 2016 software). All the tests were conducted in triplicate, and the obtained results are expressed as mean ± standard deviation. Significant statistical differences were considered *p* < 0.05.

## 4. Conclusions

In our work, for the first time, IRLIR technology was used in the extraction of bioactive compounds from *Rosa canina* L. The IRLRI technique has the advantages of short extraction time, low volume of extraction solvent used, low energy consumption, and lower costs for the extraction device, while at the same time providing a certain degree of extraction specificity if appropriate modulation of the laser source, solvents, and extraction time is employed. The optimal conditions for the IRLIR technique are as follows: 1064 nm laser source, 150 mW laser power, 15 min extraction time for both lipophilic and hydrophilic extractions, while a 20% ethanol extraction solvent was the most appropriate for polyphenol extraction. The *Rosa canina* L. extracts obtained through IRLIR with 20% ethanol exhibited significantly higher concentrations of gallic acid (131.66 µg mg^−1^) and catechin (173.37 µg mg^−1^), both of which are compounds known for their strong antioxidant activity. Gallic acid has anti-inflammatory, anti-bacterial, anti-viral, antimutagenic, anti-melanogenic, and anti-cancer activities, controlling each phase of carcinogenesis [30,43]. In addition, catechins have anti-cancer, anti-melanogenic, anti-microbial, anti-allergenic, anti-inflammatory, and anti-viral activities, present UV protection and neuroprotective properties, and can modulate lipid peroxidation [43,53]. For the extraction of ellagic acid and quercetin and its derivates, the use of 80% ethanol as the extraction solvent is the most suitable. At the same time, the amounts of carotenoids and tocopherol recovered from *Rosa canina* L. support the suitability of IRLIR technology use to recover antioxidants and anticarcinogenic lipophilic compounds from vegetal material.

The developed IRLIR extraction method is an innovative approach that supports environmental sustainability and, with appropriate upscale, can be applied at the industrial scale, providing bioactive principles for food, cosmetic, and pharmaceutical industries since it has proven its capability to preserve the efficacy of the recovered compounds, thus ensuring extracts enriched in antioxidant and anti-inflammatory compounds.

*Rosa canina* L. extracts have been demonstrated to be a rich source of natural bioactive compounds that confer antioxidant properties.

It must be stressed that IRLIR has the advantage of the potential use of more than one laser source concomitantly, thus widening the applicability range.

## Figures and Tables

**Table 1:** Some performance characteristics of the HPLC-MS method used for polyphenol compound analysis.

Compound	[M − H]^−^	t_R_, min.	Linear Regression Equations	R	Linearity Range of Response, µg mL^−1^	LoD, µg mL^−1^	LoQ, µg mL^−1^
Gallic acid	169	5.82	A = 48,686.49 × C − 4680.433	0.9993	1–100	0.16	0.30
Chlorogenic acid	353	15.18	A = 15,009.58 × C − 434.9512	0.9999	1–100	0.33	0.45
Quercetin	301	35.52	A = 72,301.05 × C – 10,684.39	0.9997	1–100	0.19	0.29
Quercitrin	447	27.78	A = 56,594.18 × C − 3157.114	0.9998	1–100	0.11	0.23
Quercetin 3-β-D-glucoside	463	25.48	A = 47,325.40 × C − 8096.301	0.9992	1–100	0.23	0.38
Rutin	609	24.81	A = 35,613.21 × C − 747.2902	0.9995	1–100	0.11	0.30
(+)-Catechin	289	15.65	A = 19,405.45 × C − 6469.358	0.9999	1–100	0.35	0.38
Ellagic acid	301	25.52	A = 58,850.16 × C – 16,277.28	0.9993	1–100	0.33	0.45

**Table 2:** The content of polyphenolic compounds obtained by the HPLC-MS method for *Rosa canina* L. extracts with 20% ethanol through IRLIR.

IRLIR, 20% Ethanol			Polyphenolic Compounds, µg g^−1^							
Laser Wavelength, nm	Laser Power, mW	Extraction Time, min.	(+)-Catechin	Rutin	Quercitrin	Quercetin	Quercetin 3-β-D-Glucoside	Gallic Acid	Ellagic Acid	Chlorogenic Acid
1064	150	5	126.13 ± 1.23	6.30 ± 0.07	25.38 ± 0.28	8.52 ± 0.08	57.51 ± 0.45	107.61 ± 0.95	24.83 ± 0.18	19.17 ± 0.15
		15	173.37 ± 1.68	6.87 ± 0.06	35.02 ± 0.31	8.98 ± 0.09	76.69 ± 0.62	131.66 ± 1.19	35.36 ± 0.24	23.07 ± 0.19
	300	5	150.7 ± 1.48	6.09 ± 0.05	31.14 ± 0.40	9.19 ± 0.09	62.37 ± 0.61	104.54 ± 0.87	28.85 ± 0.23	24.4 ± 0.21
		15	104.85 ± 0.82	6.05 ± 0.04	25.75 ± 0.32	10.64 ± 0.11	50.83 ± 0.48	98.59 ± 0.69	30.02 ± 0.29	18.21 ± 0.15
1270	150	5	135.78 ± 1.62	6.48 ± 0.06	27.36 ± 0.22	9.83 ± 0.09	63.49 ± 0.54	123.13 ± 1.09	26.67 ± 0.21	20.98 ± 0.18
		15	138.79 ± 1.37	6.03 ± 0.04	30.93 ± 0.26	9.61 ± 0.08	68.47 ± 0.61	123.65 ± 1.12	34.22 ± 0.30	19.95 ± 0.17
	300	5	138.37 ± 1.05	5.54 ± 0.04	27.68 ± 0.24	9.96 ± 0.09	55.28 ± 0.52	101.71 ± 0.85	29.25 ± 0.27	18.87 ± 0.16
		15	110.58 ± 0.85	6.22 ± 0.08	27.67 ± 0.23	10.97 ± 0.12	53.29 ± 0.48	113.30 ± 0.96	27.16 ± 0.26	19.64 ± 0.19
1550	150	5	142.34 ± 1.22	6.49 ± 0.04	27.88 ± 0.29	10.23 ± 0.10	61.04 ± 0.56	120.16 ± 1.14	28.95 ± 0.29	20.53 ± 0.20
		15	139.11 ± 1.57	6.07 ± 0.09	28.66 ± 0.27	8.68 ± 0.08	61.23 ± 0.57	112.88 ± 1.07	32.95 ± 0.29	18.28 ± 0.21
	300	5	115.07 ± 0.87	6.06 ± 0.04	29.77 ± 0.30	10.47 ± 0.11	58.33 ± 0.55	101.82 ± 1.00	34.28 ± 0.31	18.76 ± 0.18
		15	137.96 ± 1.33	5.81 ± 0.04	27.28 ± 0.25	12.83 ± 0.13	58.50 ± 0.52	112.97 ± 0.93	34.17 ± 0.32	17.29 ± 0.16

**Table 3:** The content of polyphenolic compounds obtained by the HPLC-MS method for *Rosa canina* L. extracts with 80% ethanol through IRLIR.

IRLIR, 80% Ethanol			Polyphenolic Compounds, µg g^−1^							
Laser Wavelength, nm	Laser Power, mW	Extraction Time, min.	(+)-Catechin	Rutin	Quercitrin	Quercetin	Quercetin 3-β-D-Glucoside	Gallic Acid	Ellagic Acid	Chlorogenic Acid
1064	150	5	44.00 ± 0.42	2.71 ± 0.03	47.33 ± 0.43	15.38 ± 0.15	89.06 ± 0.82	<LoD	16.72 ± 1.12	11.64 ± 0.10
		15	47.13 ± 0.49	2.65 ± 0.03	40.37 ± 0.39	14.59 ± 0.13	77.68 ± 0.79	<LoD	104.42 ± 1.03	9.00 ± 0.08
	300	5	8.02 ± 0.08	2.62 ± 0.02	38.41 ± 0.35	15.36 ± 0.15	54.89 ± 0.52	8.17 ± 0.07	107.66 ± 1.02	16.93 ± 0.14
		15	7.65 ± 0.07	2.68 ± 0.04	41.42 ± 0.40	16.44 ± 0.17	59.29 ± 0.57	8.91 ± 0.08	118.92 ± 1.12	18.38 ± 0.17
1270	150	5	42.72 ± 0.41	2.55 ± 0.02	36.42 ± 0.34	13.94 ± 0.12	71.35 ± 0.69	<LoD	99.40 ± 0.95	7.87 ± 0.06
		15	37.88 ± 0.39	2.54 ± 0.02	39.02 ± 0.38	14.20 ± 0.14	71.54 ± 0.71	<LoD	102.65 ± 0.99	8.07 ± 0.07
	300	5	7.57 ± 0.07	2.65 ± 0.02	41.43 ± 0.41	16.06 ± 0.15	57.88 ± 0.57	8.68 ± 0.07	113.49 ± 1.05	18.05 ± 0.18
		15	6.69 ± 0.06	3.14 ± 0.03	40.31 ± 0.41	15.98 ± 0.16	55.74 ± 0.52	8.57 ± 0.07	112.56 ± 1.04	17.92 ± 0.16
1550	150	5	37.72 ± 0.35	2.39 ± 0.02	35.88 ± 0.36	13.75 ± 0.11	64.49 ± 0.68	<LoD	95.88 ± 0.93	7.62 ± 0.06
		15	38.38 ± 0.41	2.62 ± 0.03	37.03 ± 0.38	15.40 ± 0.13	63.83 ± 0.62	4.12 ± 0.04	104.42 ± 1.01	11.03 ± 0.13
	300	5	13.04 ± 0.12	2.62 ± 0.02	34.36 ± 0.32	15.69 ± 0.15	42.98 ± 0.40	10.63 ± 0.09	92.46 ± 0.89	16.37 ± 0.15
		15	17.63 ± 0.15	4.28 ± 0.04	43.21 ± 0.42	16.12 ± 0.16	60.55 ± 0.58	14.43 ± 0.13	97.72 ± 0.92	15.66 ± 0.14

**Table 4:** The content of major polyphenolic compounds obtained by the HPLC-MS method for *Rosa canina* L. extracts with 20% and 80% ethanol through ASE.

Extraction Solvent	Polyphenolic Compounds, mg g^−1^							
	(+)-Catechin	Rutin	Quercitrin	Quercetin	Quercetin 3-β-D-Glucoside	Gallic Acid	Ellagic Acid	Chlorogenic Acid
20% ethanol	99.50 ± 5.59	23.00 ± 1.78	110.00 ± 2.07	31.16 ± 2.01	205.68 ± 7.09	9.52 ± 0.39	266.87 ± 14.81	25.11 ± 0.18
80% ethanol	60.44 ± 6.58	11.14 ± 0.42	63.75 ± 4.14	27.56 ± 0.63	70.20 ± 5.05	<LoD	160.54 ± 10.08	15.25 ± 0.75

**Table 5:** Some performance characteristics of the HPLC-DAD method used for lipophilic compound analysis.

Compound	t_R_, min.	Linear Regression Equations	R	Linearity Range of Response, µg mL^−1^	LoD, µg mL^−1^	LoQ, µg mL^−1^
α-tocoferol	4.91	A =13,185 × C − 7529	0.9976	5–115	2.20	3.25
β-caroten	6.44	A = 5313 × C − 27752	0.9983	45–275	13.10	28.20

**Table 6:** The content of lipophilic compounds obtained by the HPLC-DAD method in *Rosa canina* L. extracts through IRLIR.

IRLIR			Lipophilic Compounds, µg g^−1^			
Laser Wavelength, nm	Laser Power, mW	Extraction Time, min.	α-Tocopherol	β-Carotene	Lycopene	Lutein
1064	150	5	251.04	1595.79	1627.08	414.56
		15	283.23	1804.23	1892.33	450.09
	300	5	258.36	1708.86	1852.18	429.94
		15	255.70	1716.57	1808.09	436.92

**Table 7:** TEAC values for *Rosa canina* L. polyphenolic extracts obtained with ABTS.

Extract Solvent	Laser Wavelength, nm	Laser Power, mW	Extraction Time, min.	TEAC, µmol g^−1^
20% ethanol	1064	150	5	23,456 ± 55
			15	22,672 ± 48
		300	5	17,492 ± 384
			15	21,825 ± 154
	1270	150	5	23,488 ± 48
			15	22,289 ± 315
		300	5	20,114 ± 138
			15	21,441 ± 154
	1550	150	5	22,401 ± 182
			15	23,619 ± 305
		300	5	20,786 ± 499
			15	22,193 ± 584
80% ethanol	1064	150	5	5238 ± 44
			15	3330 ± 194
		300	5	3791 ± 75
			15	4331 ± 82
	1270	150	5	4650 ± 168
			15	3370 ± 52
		300	5	3863 ± 52
			15	4225 ± 50
	1550	150	5	4656 ± 102
			15	3462 ± 99
		300	5	4100 ± 142
			15	3988 ± 97

**Table 8:** Antioxidant capacity values for *Rosa canina* L. polyphenolic extract obtained with ORAC.

Laser Wavelength, nm	Laser Power, mW	Extraction Time, min.	TEAC, µmol g^−1^
1064	150	5	31,701 ± 63
		15	117,260 ± 235
	300	5	68,872 ± 138
		15	33,398 ± 67
1270	150	5	9855 ± 20
		15	100,944 ± 202
	300	5	55,465 ± 111
		15	11,828 ± 24
1550	150	5	22,117 ± 44
		15	53,392 ± 107
	300	5	33,722 ± 67
		15	32,619 ± 65

**Table 9:** TEAC values for *Rosa canina* L. polyphenolic extract obtained through ASE.

Extract Solvent	TEAC (ABTS), µmol g^−1^	TEAC (ORAC), µmol g^−1^
20%	14,699 ± 282	19,707 ± 158
80%	1299.3 ± 5.7	10,058 ± 57

**Table 10:** Antioxidant capacity values for *Rosa canina* L. lipophilic extract obtained with ORAC assay.

Laser Wavelength, nm	Laser Power, mW	Extraction Time, min.	AC, µmol g^−1^
1064	150	5	6881 ± 33
		15	8475 ± 35
	300	5	6202 ± 28
		15 min	6353 ± 18

**Table 11:** Laser sources characteristics, according to the technical sheet from the supplier.

Parameters	Characteristics		
Wavelength, nm	1064 ± 1	1270 ± 10	1550 ± 20
Operating mode	Continuous	Continuous	Continuous
Output power before fiber, W	1.637	1.275	1.246
Output power after fiber, W	1.511	1.100	1.100
Power control, %	0–100	0–100	0–100
Power stability, %	<3	<3	<3
Warm-up time, min.	<10	<5	<5
Fiber connecter	SMA905	SMA905	SMA905
Fiber, um@1 m	400	400	400
Beam dimension, mm	~5 × 8	~5 × 8	~5 × 8
Beam divergence, mrad	<1.5	<3.0	<3.0
Beam diameter at 1/e^2^, mm	~1.5	~1.5	~1.5
Spot diameter after adding end collimator @10 cm, mm	~35	~35	~35
Beam high from base plate, mm	24.8	29	24.8
Operating temperature, °С	10~35	10~35	10~35

**Table 12:** Operational parameters of the laser radiation during the extraction process.

Wavelength, nm	Current, A/Power, mW	Energy Consumption, kW
1064	0.71/150	0.020–0.030
	0.97/300	
1270	0.9/150	0.029–0.030
	1.22/300	
1550	0.80/150	0.037–0.038
	1.22/300	

## Data Availability

The authors confirm that the data supporting the findings of this study are available within the article.
